# The influence of night length: Activity of the northern bat *Eptesicus nilssonii* under conditions of continuous light in midnight sun compared to a southern population

**DOI:** 10.1186/s40850-021-00099-1

**Published:** 2021-12-13

**Authors:** Karl Frafjord

**Affiliations:** Tromsø University Museum, UiT The Arctic University of Norway, P.O. Box 6050 Langnes, 9037 Tromsø, Norway

**Keywords:** Light level tolerance, Limit of distribution, Midnight sun, Nightly activity, North-south gradient, Seasonal activity

## Abstract

**Background:**

Nearly all insectivorous bats (Chiroptera) are strictly nocturnal, flying and feeding only between sunset and sunrise despite lower insect availability than by day, most likely to avoid predation by diurnal birds. This may represent a great challenge to bats living north of the Arctic Circle, which are exposed to bright nights in the period of the midnight sun. The northern bat *Eptesicus nilssonii* was studied at different latitudes in Norway (69, 66 and 58°N) by three techniques; visual counts of exits from and returns to roosts, infrared detection with a datalogger and an ultrasound data recorder, to reveal how their activity varied across latitude, season, and night, as well as across light levels. How does a nocturnal bat adjust to perpetual light and what light levels are tolerated?

**Results:**

In the north the bats’ active season lasted 2.5 months, 1.5 months shorter than in the south. The bats only flew in 3-4 weeks of midnight sun, and hardly ever left the roost until the sun went behind a hill in the evening. In addition, the timing of their nightly hunting was highly influenced by the darkness of the sky, and they very rarely flew in light levels above 200 foot-candles (FC). As the night became darker than twilight from early August, the bats restricted their activity to between sunset and sunrise. This was the normal situation in southern Norway, where the bats tracked sunset and sunrise throughout the entire season. Those bats appeared to prefer light levels below 100-50 FC and hence, also did fly in twilight conditions.

**Conclusions:**

The willingness to fly in twilight by the southern population may be a prerequisite to the northern bat’s survival in the land of the midnight sun. These bats must accept short nights in the first part of their summer season and must be willing to fly in light levels 2-4 times higher than in the south. Most likely, this depends on a reduced predation risk and good abundance of insects at night.

## Background

Nearly all insectivorous bats (Chiroptera) are strictly nocturnal, flying and feeding only between sunset and sunrise [1–3]. The key factor for their preference for nocturnal flying seems to be predation risk [1, 2, 4–7], caused by diurnal predators (raptors, even corvids and other birds) being more numerous or successful in hunting bats than nocturnal predators (owls, that rarely pursue flying prey). A few species may start to fly before sunset, this is thought to be associated with reduced predation risk, such as for the Azorean bat *Nyctalus azoreum* [8, 9]. Competition with birds for insect prey or air space may also influence the timing of hunting in bats [1] and thermal constraints of daylight flight may also explain why bats became nocturnal [10, 11].

Most insect species are on the other hand, more active by day than by night [12, 13]. Consequently, the nocturnal hunting of bats may not seem to be an optimal feeding strategy [1]. Some insect groups, particularly Diptera and moths (Lepidoptera), are to a larger extent nocturnal and can, at least in some circumstances, be very numerous [5, 14]. The northern bat *Eptesicus nilssonii* is widely distributed in northern Eurasia and is, in Europe, distributed between appr. 44-69°N [15–17]. In central Europe, northern bats hibernate from November to April [18, 19]. They arrive at nursery roosts in early May, and parturition occurs in the last half of June or in July [12]. The roosts are abandoned in late August to early September, perhaps even in late July [18, 19].

Going north in the temperate region, the summer nights become shorter and brighter, eventually leading to perpetual bright nights under the midnight sun north of the Arctic Circle. The only bat breeding substantially north of the Arctic Circle is the northern bat [6, 14–16]. At this latitude, the summer lasts only 2-3 months, during which female bats go through the energetically demanding periods of gestation and lactation. The young must grow quickly and deposit a fat layer to be able to survive the 9-10 months of hibernation. Such a short “window” for reproduction, combined with perpetual light in the period of midnight sun, could be a recipe for disaster. Hunting in bright nights must be a trade-off between avoiding predators and the need to feed. Extending hibernation until the night becomes dark is no option with such a short season. The willingness by an otherwise nocturnal species to fly under the midnight sun is very interesting. The exit (emergence) time is of particular interest because the bats may aim to take advantage of a higher abundance of insects in the early evening (the evening peak), but then they also run higher risk of predation [5, 20].

The main aim of this study was to describe the activity of the northern bat under continuous light in midnight sun. The hypotheses are that this nocturnal species tolerates relatively high levels of light intensity, that it is still limited to hunt within certain limits of light intensity, that the midnight sun poses a limitation, and that increasing latitude decreases the amount of time available for hunting. The following questions and predictions will be investigated. How does the daily and seasonal activity in the land of the midnight sun compare to locations further south? How accurately does the species track changes in night length and adjust its activity to both short- and long-term changes in light levels? Predictions include: The active (non-hibernating) season is much shorter in the north than in the south. In the south, the bats adjust their nightly flying as the season progresses, principally to between sunset and sunrise. In the bright nights of the midnight sun period in the north, the bats restrict their flying to the darkest hours of the night. A south-north gradient in the length of night-time foraging and hence, light tolerance, can be found.

## Results

### Activity

**Fig. 1:**
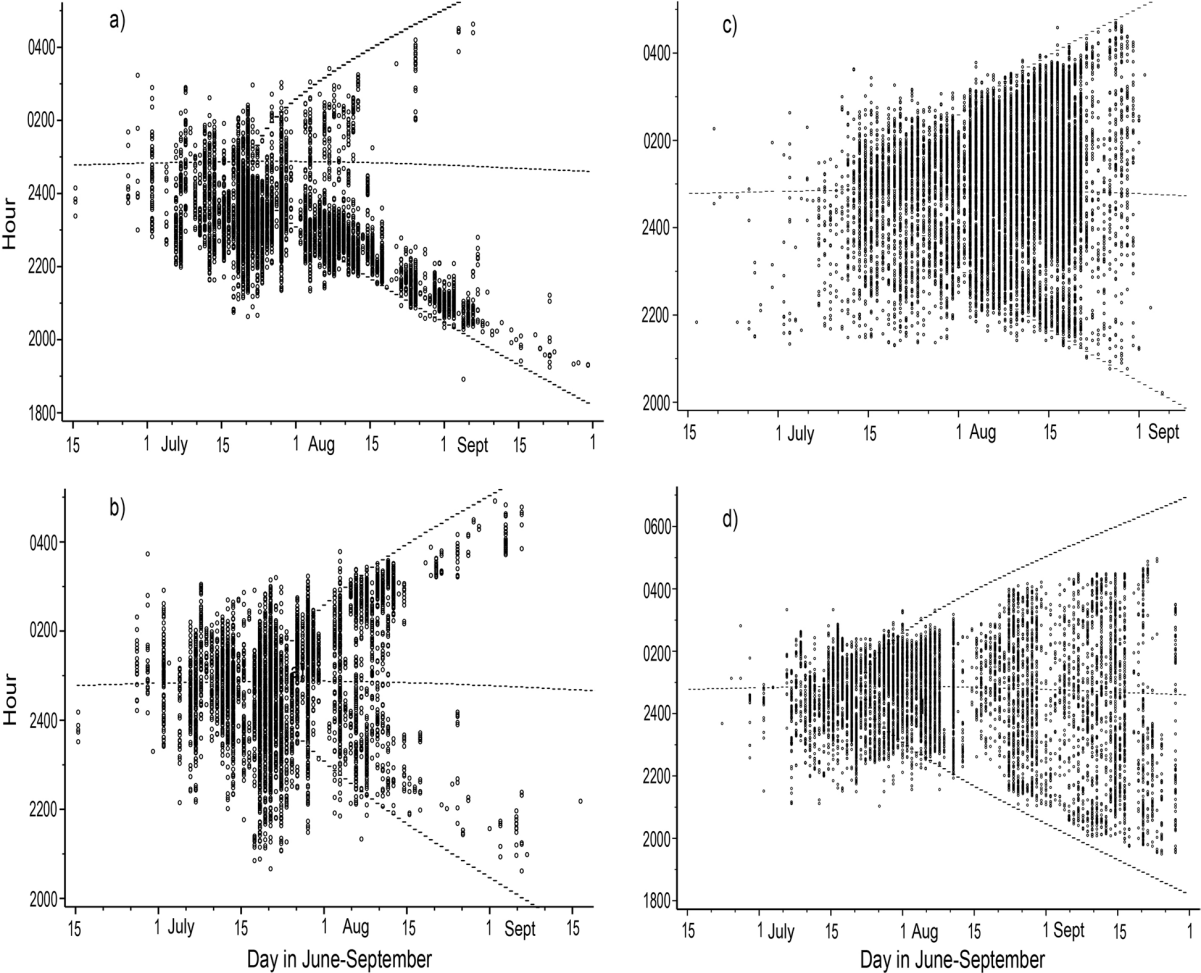
Distribution of a) roost exits and b) roost returns by the northern bat during the night across the whole season. Recordings made by visual counts from mid-June to the end of September at all roosts in Troms (including Løvhaug). Distribution of northern bat passes during the night across the season. c) Recordings made by IR detection in three summers (mid-June to early September) at the roost Løvhaug (a few records from September and October are not included). d) Ultrasound recordings (late June to the end of September) in Troms. The lower dashed line represents sunset, the upper represents sunrise and the central represents the sun at its lowest angle. Note that many points are superimposed

**Fig. 2:**
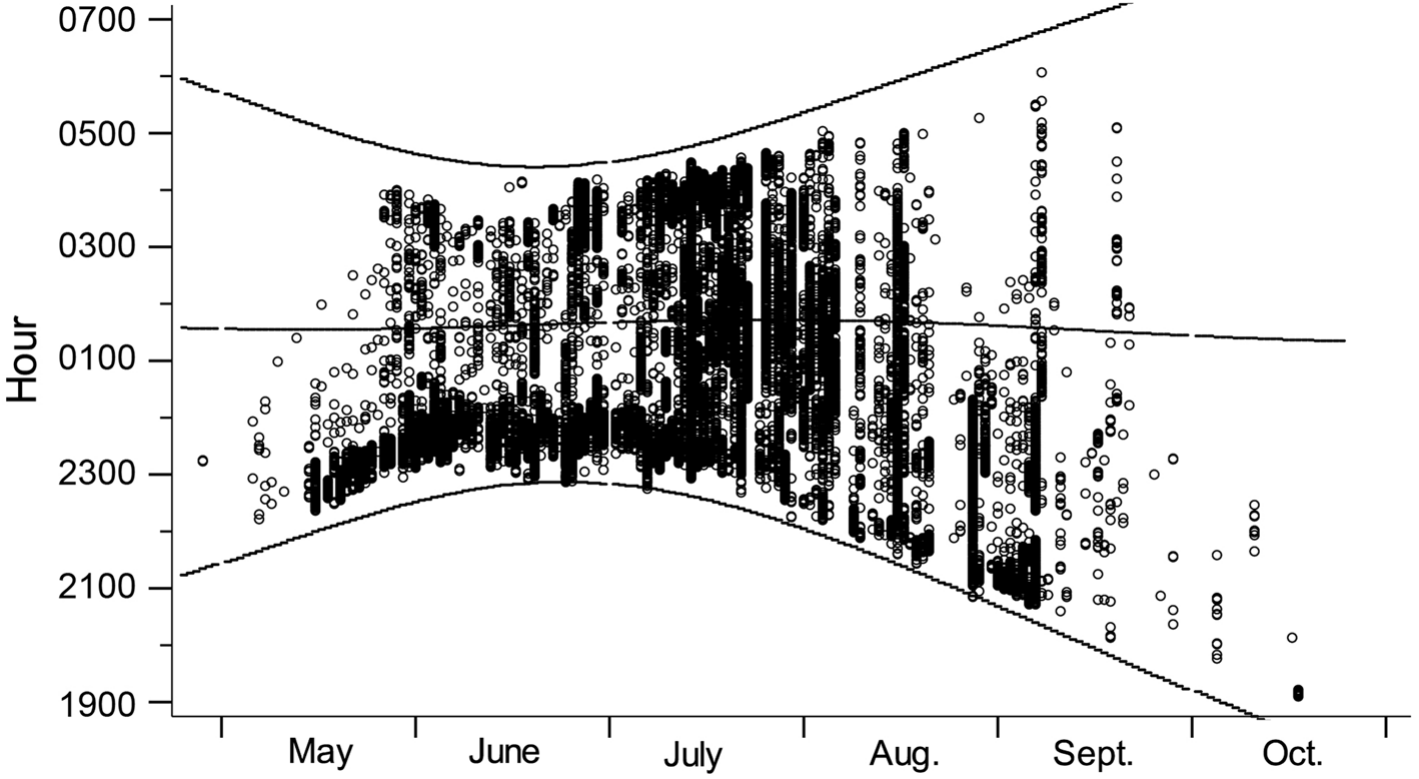
Distribution of northern bat passes recorded by ultrasound during the night across the whole season at the main site in Frafjord (n=10 489 minutes, late April to mid-October). The lower dashed line represents sunset, the upper represents sunrise and the central represents the sun at its lowest angle. Note that many points are superimposed. From the last days of September to October ultrasound recordings were only made in the first half of the night

In Troms, most activity was recorded between late June and early September (Fig. 1), with the most extreme records made 6 June and 16 October. In Saltdal, the earliest record was made 8 June (three bats), and the main active period started 6 d earlier than in Troms. In Frafjord, the earliest records were made 28 April 2018 and 20 April 2020 and the last 17 October 2018. Here, a large peak in activity was found during 11-20 July (Fig. 2).

The bats’ nightly activity was almost perfectly centered around the time when the sun was at its lowest angle (SLA, Figs. 1-2, Table 1 gives the number of recordings). In Troms, visual counts showed a large overlap in the timing of exits and returns (Fig. 1a-b). During the period of midnight sun, the timing of exits and returns varied much (Fig. 1a-b). In the midnight sun period, the activity was still centered around SLA, but the bats regulated their exits and returns to a large extent according to the darkness of the sky. This continued somewhat after the end of the midnight sun period, to around 10 August when the night started to become proper dark. Thereafter, the bats limited their activity almost exclusively to between sunset and sunrise (Fig. 1a-b).

**Table 1:** Number of recordings by the three different recording techniques used at various roosts and sites

Roost/Region	Technique	Exits	Returns	Passes	Calls	Minutes	Nights
Løvhaug	Visual counts	6578	4181				123
Troms, excl. L. Løvhaug	Visual counts	3283	1891				162
Saltdal [2]	Visual counts	648	746				24
Løvhaug	IR detection			26723			174
Bardu	IR detection			2465			49
Saltdal [1]	IR detection			6121			65
Troms, all sites	Ultrasound				204680		250
Frafjord, all sites	Ultrasound					12180	385

Løvhaug and Bardu are single roosts, Saltdal is either 1 or 2 roosts, Troms is multiple roosts, Frafjord represents multiple hunting sites. Nights is the number of nights the technique was in operation

IR detection and ultrasound recordings largely replicated nightly activity patterns observed with visual counts data (Fig. 1c-d). They also confirmed that the activity was continuous throughout the night and did not occur in discrete exit and return periods. IR detection showed an even more variable pattern in the midnight sun period at the Løvhaug roost (Fig. 1c), but somewhat less in the Bardu roost. In August, the activity period of the Bardu roost increased with the length of the night, with just a few exits before sunset. In June at Løvhaug, exits started mostly within one hour of SLA and in the first half of July within two hours of SLA. In the latter half of July, a wider range of exits relative to SLA was found, mostly starting within three hours of SLA (Fig. 1a). Returns to the roost showed a similar response (Fig. 1b).

In Saltdal, the bats appeared to limit their activity to between sunset and sunrise already from the middle of July. From then on relatively few visual recordings were made before sunset and after sunrise. The activity in Saltdal was even more centered around SLA than in Troms. In Frafjord, the activity throughout the season tracked sunset and sunrise very well (Fig. 2). The duration of nightly activity was shortest around mid-June (five hours), when the night is shortest. It was centered around SLA and only slightly skewed toward sunset (SLA in Frafjord was nearly one hour later than in Troms). Very few ultrasound recordings were made before sunset and none after sunrise (Fig. 2). Most intense activity was recorded during the first hour after sunset.

### Light levels at first exit and last return

**Fig. 3:**
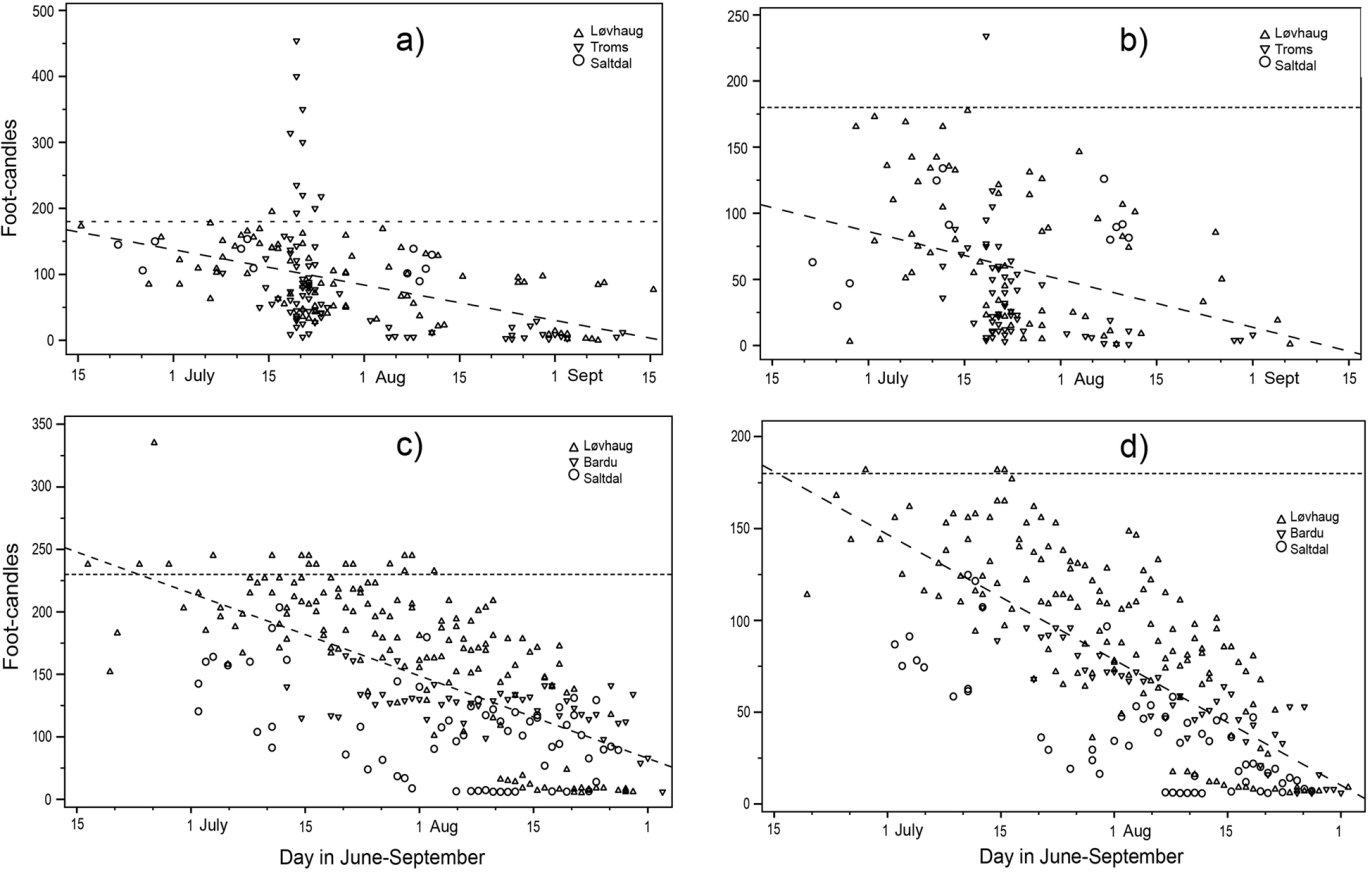
Light levels (foot-candles) at the time of first exit and last return from or to the roosts across the season. a) Recordings made at first exit (r= -0.44, p<0.001, n=176) and b) at last return (r=-0.34, p<0.001, n=135) by visual counts at Løvhaug, all other roosts in Troms and at two roosts in Saltdal. c) Recordings made at the time of the first (r=-0.64, p<0.001, n=263) and d) last pass (r=-0.74, p<0.001, n=244) by IR detection at the three roosts Løvhaug, Bardu and Saltdal. The horizontal lightly dashed line represents 180 foot-candles, and the heavier dashed line is the regression line

Visual counts at all roosts in Troms and Saltdal revealed that the bats very rarely exited in light levels above 180 foot-candles (FC, Fig. 3a and b). A few exceptions up to 480 FC were recorded during the annual counts in July, most being from the same roost (where IR detection was not used). Levels of light at first exit decreased during the season, to below 100 FC from the middle of August, similarly in the three groups Saltdal, Løvhaug and Troms (Fig. 3a). With only one exception, the northern bat always returned to the roost at light levels below 180 FC (Fig. 3b).

Light levels logged simultaneously with IR detection in operation were similar to those recorded during visual counts (Fig. 3). Nearly all first exits (movements) occurred in light levels below 200 FC and most below 180 FC (Fig. 3c). Note that four recordings up to 2200 FC during short bursts of sunshine from a cloudy sky were excluded from Fig. 3c. Nearly all last returns were in levels below 180 FC and at slightly lower levels than at first exit (Fig. 3d). The seasonal trends of diminishing light levels at both exit and return were notably similar, approaching 0 FC in late August.

With IR detection, significant differences in light levels were found between the three roosts, at both first exit (one-way ANOVA with Tukey test: F=46.4, d.f.=2, 260, p<0.001) and last return (F=48,1, d.f.=2, 240, p<0.001) in July and August. The passes at Løvhaug were made at higher levels (exit mean=119.2, return mean=95.5 FC, paired samples test: t=8.63, d.f.=132, p<0.001) than both in Bardu (exit=74.4, return=56.0, t=5.56, d.f.= 45, p<0.001) and in Saltdal (exit=54.2, return=37.8, t=6.24, d.f.=53, p<0.001).

Visual counts gave similar results. At the single roost Løvhaug, mean light level at first exit was 103.7±49.4 and at last return 79.9±54.5 FC (t=4.94, d.f.=50, p<0.001). At the other roosts in Troms combined, light at exit was 98.8±94.4 and at return 35.9±41.6 FC (t=4.66, d.f.=43, p<0.001). At the two roosts in Saltdal, light at exit was 124.6±22.2 and at return 87.2±32.7 FC (t=3.18, d.f.=10, p=0.01). Overall, light levels at return were 61 and 77% of those at exit, as recorded by visual counts and IR detection, respectively.

**Fig. 4:**
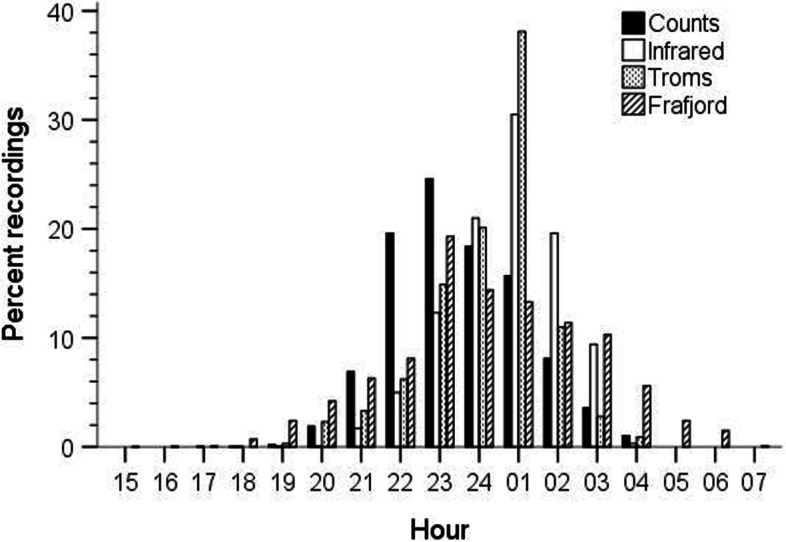
Percent recordings by hour and different recording techniques. Counts: visual counts (both exits and returns) at all roosts in Troms, Infrared: IR detection at two roosts in Troms, Troms: ultrasound recordings, Frafjord: ultrasound recordings

Light level recordings from Sandnes were used to explore the expected order of magnitude the levels in Frafjord could be. In Frafjord, most first bat passes were recorded between 2200 and 2359 h and most last passes between 0400 and 0559 (Fig. 4). Light levels at 22 and 05 hours and at 23 and 04 hours were almost identical, hence only the first part of the night was examined. During the four hours 22 to 01 prior to SLA, the maximum light levels at Sandnes always were below 110 FC and from 1 July always below 100 FC. At 23 h the maximum rarely exceeded 50 FC. Mean FC at 2200 h was 64.5±32.0 and at 2300 h 20.0±15.9.

For comparison and as an example, light levels measured at Løvhaug in 2002 were explored in the same way. These maximum levels were constantly higher than those in Sandnes until late August, when levels at both sites approached zero early in the evening. During the season, the light levels at the bats’ first exit closely tracked the maximum levels measured at 22 h. However, in early August the bats switched rather abruptly to near zero light, i.e., earlier than the maximum light levels at 22 h. At 23 h in Sandnes, the level approached zero in the middle of July, compared to early August at Løvhaug.

### Duration of activity

Nearly all activity was recorded between 2200 and 0359 hours (Fig. 4). The overall proportion of visual counts were skewed to before SLA (Fig. 4) due to the priority given to counting bats emerging from the roosts. In Troms, the exit period (from the first exit until numbers of returns exceeded those of exits) lasted overall 74.0±40.1 min, maximum 216 min: (1.8 bats min^-1^ with exits). The first 25% of the numbers in the roost took 27.9±23.6 min to exit, 50% took 42.2±28.3 min and 75% took 57.3±33.7 min.

The return period (from the time when numbers of returns exceeded those of exits until the last bat returned) lasted longer than the exit period (107.6±62.4 min, maximum 350 min, 1.4 bats min^-1^ with returns). The first 25% of the number of bats took 31.4±33.3 minutes, 50% took 54.6±50.2 minutes and 75% took 75.4±53.0 minutes. The period of exit was much shorter on dark than on bright nights. Data from Troms were arranged into dark (<100 FC) and bright (≥100 FC) nights. During dark nights, the time to reach 25, 50, 75 and 100% of the exits was 56-67% of the time taken during bright nights. The duration of 100% exits was 66.1 min during dark nights vs. 98.7 min for bright nights (F=24.4, d.f.=1, 121, p<0.001). There was no statistically significant difference between the durations of returns on dark vs. bright nights.

The estimated maximum times between the first exit and the last return were similar when using the three techniques and increased with the season from around four hours in June to almost ten hours in September (Table 2). The only significant difference was between visual counts at the roost Løvhaug and ultrasound recordings in Frafjord (Table 2, independent samples test: t=3.9, d.f.=6 time periods, p<0.01, a “false discovery rate” control did not change the conclusions).

**Table 2:** Duration of maximum activity periods (minutes) in 10-days intervals as recorded by three different recording techniques

Time	IR detection			Visual counts			Ultrasound rec.	
period	Løvhaug	Bardu	Saltdal	Løvhaug	Troms	Saltdal	Troms	Frafjord
11-20 May								248
21-31 May								316
1-10 June								286
11-20 June								272
21-30 June	275			244			254	313
1-10 July	299		337	304	167	240	433	312
11-20 July	368	223	300	314	370	257	433	338
21-31 July	348	242	326	356	394	285	357	384
1-10 Aug.	376	357	330	387	323		345	429
11-20 Aug.	440	405	470	361	465	454	315	454
21-31 Aug.	388	416	501	470	508	439	430	505
1-10 Sept.				595		459	513	568
11-20 Sept.							524	538
21-30 Sept.							568	

The techniques were: IR detection at the roosts Løvhaug, Bardu and Saltdal, visual counts at the roost Løvhaug, at all other roosts in Troms combined and at two roosts in Saltdal, and ultrasound recordings in Troms and in Frafjord. The duration is the time between the first exit/pass and the last return/pass

### Temperatures at exit and return

An example of mean monthly temperatures (in 2002) is given in Table 3. Minimum temperatures recorded at Løvhaug in 2002 were -1.0 °C in June, 3.7 °C in July and 0.7 °C in August. During IR detection, the mean temperature at first exit was 11.6±3.3 °C, minimum 3.1 and maximum 21.1 (n=263, all three roosts combined). The mean temperature at last return was 9.5±3.6 °C, minimum -2.5 and maximum 18.1 (n=244). The differences between temperatures at exit and return were statistically significant (t=15.4, d.f.=237, p<0.001). During visual counts, the mean temperature at first exit was 11.5±9.4 °C, minimum 2.1 and maximum 21.0 (n=208). At the last return the mean was 9.4±3.6 °C, minimum 0.0, maximum 19.0 (n=165). Again, the difference between temperatures at exit and return was significant (t=12.7, d.f.=127, p<0.001).

**Table 3:** Mean monthly temperatures recorded at the roost Løvhaug in 2002, including temperatures at exit and return

Month	Mean	First exit	Last return	Bardufoss
June	12.9	11.2	8.8	13.7
July	15.8	13.6	10.3	14.6
Aug.	14.5	10.0	9.4	13.9
Sept.	9.7			6.6
Oct.				-2.6

Temperatures were recorded with a data logger at Løvhaug (16 June – 5 September 2002, n=23 239 records). Both monthly means and temperatures at first exit (n=62) and last return (n=55) during the same period are shown. Monthly means for 2002 from the official weather station at Bardufoss airport are included for comparison

## Discussion

In this study, the activity of the northern bat was studied at three different latitudes; 58, 66 and 69°N. Large differences in activity were found between the two extreme latitudes. Bats were active in the far south for 1.5 months longer than bats in the north. In the far north, the bats’ season only lasted 2-3 months, during which females go through the high demands of gestation and lactation (mating may also take place in this period). At the three latitudes, the bats adjusted their activity to the progression of the season as expected, hence, a gradient from south to north is likely to exist in this respect. The timing of the active (non-hibernating) season at different latitudes results from ambient temperatures, which also affects insect abundance. Both the hypotheses and the predictions were mostly confirmed, as the bats tracked the changing sunset and sunrise well across the season. However, in the midnight sun period the timing of the bats’ flying activity varied more than could be expected, caused by the sky’s varying darkness. In this period, a bright sky did limit the bat’s hunting time, as predicted, due to the bats’ maximum limit of light tolerance.

In Frafjord and further south in Europe [12, 18, 19], the first young flew only 2-3 weeks earlier than in Troms. Apparently, the northern bat can complete reproduction in just 1-1.5 months, as inferred from the timing of volant young. A large variation in the timing of parturition (at any latitude), perhaps as much as one month between the extreme dates, would obscure any trends related to the suggested reduced activity in late pregnancy and increased activity in late lactation [12, 21]. The most significant difference found was that the bats in the south had a much longer pre-reproduction period than in the north. This may potentially increase their reproductive success, by giving them more time to improve body condition before parturition.

Only one case of attempted predation was seen during this study [9], after which the bats postponed emergence by two hours. This would probably be detrimental if prolonged for many days, as many days without sufficient foraging might jeopardize the survival of the offspring or surviving the following winter hibernation [6, 12]. With climate change, one effect of an anticipated earlier spring might be earlier and increased bat activity in June, which may possibly lead to higher reproductive success. However, this would also expose the bats to a longer period of midnight sun, and hypothetically, increased predation risk. In Troms, the sun goes below the horizon from 23 July, when the sun is below the horizon for 3 h 31 min and 6 h 46 min in Saltdal and Frafjord, respectively. Already on 31 July in Troms, the sun is below the horizon for 3 h 28 min, when the bats started to restrict their flying to between sunset and sunrise. In Sweden, a similar increase in foraging time with season was found, with the most significant increase in August [22].

In the far south, flying shortly after sunset and shortly before sunrise meant that the bats readily accepted twilight conditions [6]. These bats sometimes must have accepted light levels between 50-100 foot-candles (twilight), although probably preferred levels below 50 FC. Similar levels were reported in a study at 62°N in south-western Norway [23]. Even during the shortest night of the year in Frafjord, the bats could hunt five hours. As predicted, this was dramatically different during the period of the midnight sun in the north, where the bats accepted much higher light levels but still hunted less than two hours on the brightest nights.

The northern bat flew early in the evening compared to most other bat species in Europe [5, 21, 23–33]. The northern bat of the far north probably attempted to take some advantage of the evening peak in insects [6, 12, 34, 35], by tolerating higher light levels at first exit than at last return. The duration of the nightly activity increased throughout the season, up to nearly ten hours in the autumn (compare [36]), which may be attributed both to increased night length, reduced insect abundance and the need to fatten-up in preparation for hibernation.

## Conclusions

This is the first comprehensive attempt to study the flight activity of the northern bat across the full season, especially at the species’ northern limit of distribution. The bats’ activity and light tolerance in the far north were compared to a southern population, which may represent the most common situation for this species. The active (non-hibernating) season was much shorter in the northern population than in the southern. In the far south, the bats were only flying between sunset and sunrise, adjusting their activity to the changing night length across the season, centered around when the sun was at its lowest angle. The bats of the far north tolerated much higher light levels, regularly up to 200 foot-candles or 2-4 times more than in the south, to be able to fly in the period of the midnight sun. Despite this, the bats still restricted their activity to the darkest period of the night when the sun was hidden from view. As the night became longer and darker than twilight from August, the bats restricted flying to between sunset and sunrise also at the northern limit of their distribution (when the night lasted around 5 h). This all indicates a gradient in the tolerance of brighter nights from south to north, following a decreasing night length. The northern bat is able to survive and reproduce well north of the Arctic Circle in Norway, by its willingness to fly in brighter nights than most other bats.

## Methods

### Study sites

This study utilizes data from three different study sites and three different techniques. The sites were distributed from north to south in Norway, which enables a comparison between different light regimes and night lengths.

a) Troms county (69° 00’N 19° 00’E): Data were collected at 12 roosts with most of the effort being directed at one large roost at Løvhaug (near Rundhaug) in Troms. Bats were counted in mean 6.2±1.7 roosts per year (counted regularly during 20-30 July, leading to increased sample size in this period). Colony size ranged from 5 to > 100. The Troms population was studied between 1999 and 2019. The sun is above the horizon all 24 h (midnight sun) between 21 May and 22 July.

b) Saltdal (66° 52’N 15° 18’E): This is a large valley running N-S in Nordland county, just north of the Arctic Circle. The study site was far south in the valley. Two nearby roosts were studied in 1999-2000 and 2005-2006, and the data from visual counts were combined. The sun is above the horizon all 24 h between 27 May and 16 July.

c) Frafjord (58° 50’N 06° 18’E): This is a small valley with steep sides stretching west-east in Rogaland county, on the SW coast of Norway. It has a coastal climate with much precipitation, mild winters, and little snow. The study site was on a farm in the central part of the valley, in 2018-2019. At 58°N, Frafjord is approximately half-way between the southern and northern limits of the species’ distribution [17].

All three study sites could be characterized as rural farmland regions, with extensive farmed fields in the bottom of the valleys. In Troms and Saltdal, extensive areas were covered by forest, mainly pine *Pinus sylvéstris* and birch *Betula pubéscens*. In Frafjord, the hillsides were covered in deciduous forest with many different tree species.

### Bat recording techniques

Three different techniques were used to record bats. These supplemented each other very well, gave slightly different data and had different inherent strengths and weaknesses. There were large differences in the number of recordings made by the three techniques (Table 1).

1. Visual counts at roosts. Numbers of bats exiting and leaving or returning and entering the roost house were counted by visual observations with the aid of a heterodyne bat detector (Petterson D120 or D200). Recordings were made to the nearest minute. In Troms, visual counts were used in a long-term surveillance of bat numbers. These special recordings were made on 20-28 July, the period when most adult bats had arrived and before the young became volant. Otherwise, visual counts were made opportunistically throughout the summer.

More visual counts were made at one specific roost, Løvhaug, than at any other (Table 1). This included counting bats at various times during the whole season, both to study their activity and their seasonal arrival and departure. Particularly at Løvhaug, but also at some of the other roosts, recordings were made throughout the night on several occasions, i.e., all bats that exited or returned were counted. Counting became increasingly difficult from mid-August, as nights became too dark to see the bats. From early August until early September visual counts were mainly made during the exit period and occasionally in the return period. Only a few visual counts were made in September and October when most bats where hibernating (Frafjord 2012).

2) IR (infrared) detection. This technique was based on an infrared light beam (TrailMaster 1500 Bat) that registered the number of passes. This gave the number of movements across the beam and not the direction of the movement. The use of this technique mostly ended in early September, in one year in October, when few or no passes were recorded.

In Troms, IR detection was used at two roosts, Løvhaug (2001, 2002 and 2003) and Bardu (2004). At Løvhaug, the IR beam only covered two of about ten entrances (Table 1). At the Bardu roost (only 9 bats), the IR beam covered the main entrance used by nearly all bats. At the roost in Saltdal (2005 and 2006), the IR beam covered the full longitudinal slit used by the bats [37].

3) Ultrasound recordings. Using the ultrasound datalogger Wildlife Acoustics Song Meter2 Bat (discontinued), passes were recorded on memory cards and analysed with the software Wildlife Acoustics Song Scope (discontinued, a user guide can be found at https://www.wildlifeacoustics.com/uploads/user-guides/Song-Scope-Users-Manual.pdf). Ultrasound recordings were made in Troms from 2011 to 2017 and in 2019, the logger either was placed at or near a roost. These recordings continued until the end of September.

In Frafjord, the ultrasound recorder was used more or less continuously from 26 April 2018 to 27 July 2019 at one site, at the house of a farm. Data are missing from April and May 2019 due to a faulty memory card, hence, this period was covered in 2020 (21 March to 16 June), but these data were here only used to find the start of the bat’s season and are not otherwise included. The microphone was placed just outside the house towards the garden and about 3 m above ground. In the summers of 2017 and 2019, the recorder was also used at other sites in the valley for a few weeks to give supplemental data.

Generally, in both Troms and Frafjord, the recorder ran from 0.5 or 1.0 h before sunset and until the same interval after sunrise. From October to December 2018, the recording period in Frafjord was reduced to around nine hours from sunset. This was done to limit the huge work of going through all the files generated. In Troms, with only one species of bat, there was no need to identify species, but noise recordings (mostly rain and wind) were eliminated from the output file. All 30-min data-files were checked, but not every single sound in a file. The resulting output file consisted of all ultrasound emissions from northern bat passes that were identified by the software, normally only some of the sounds produced during a bat pass. Simultaneous recordings of multiple bats were not accounted for.

In Frafjord, eight bat species are known, so the species recorded had to be identified, a much more elaborate procedure. Because most output files contained several species (often passing simultaneously), they could not be used directly as in Troms. Instead, a new file was made based on every minute a northern bat was recorded, so the sample size is the number of minutes and not the number of ultrasounds/passes (Table 1).

### Weather and light data

During visual counts, a hand-held light meter, Extech Instruments 401027, was used to measure light intensity in foot-candles (abbreviated FC, 1 FC=10.764 lux), with the sensor pointed toward the brightest part of the sky. Readings were taken specifically when the first bat exited the roost and the last bat returned. A thermometer was used to determine the temperature at the same time, 1-2 m above ground.

When the TrailMaster was operating, a Pace Scientific Inc. Pocket logger XR440 was used to measure and record light intensity (in FC) and temperature. The light sensor was mounted on top of a 3 m pole placed in open space close to the roost. The temperature sensor was mounted just below the light sensor in the opening of a white, everted plastic container with open bottom. Recordings were made every five minutes through all 24 hours.

Data on light and temperature were not collected in Frafjord, but some data on light levels collected in the nearby city Sandnes (58° 51’N 5° 44’E) in 2007 were used for comparison. These data were collected by the Pocket logger XR440 as described above, with the sensors placed about 5 m above the ground. One recording was made every 10 min throughout the 24 h. I used maximum number of FC recorded during an hour, for example 22 h = 2200-2259 h.

### Definitions and analyses

The term “activity” is used for bats flying outside their roost, measured either by the timing of exits and returns or by the number of passes throughout the night. This is a measure of roost or population activity, not the activity of individual bats. A bat may return to the roost several times during the night, which can be illustrated by the number of exits and returns or passes. The period between the first exit and the last return of bats in the roost is the groups maximum activity period.

The timing of the sunset, sunrise, and sun at its lowest inclination angle (SLA) in the north was download from https://www.timeanddate.no/ (September 2019). Statistics for Setermoen (within the study area) were used for Troms, from Rognan (25 km north of the study area) for Saltdal, and from Sandnes (32 km west of the study area) for Frafjord. Because sunset and sunrise are only two minutes earlier in Frafjord than in Sandnes, this small difference was ignored.

The exit period was defined as the time from the first bat exit to the time when numbers of returns exceeded the numbers of exits. The return period was defined as the period from when the number of returns exceeded the number of exits to the last return. All data were adjusted to Norwegian summertime (daylight saving time, UTC+2 h). Because bat activity was centered around midnight, the presentations are based on “night” rather than “day”. Each night crosses two dates but was assigned to its start date.

Statistical tests include the paired and independent samples t-test (t), one-way ANOVA (F) with Tukey post hoc HSD test and Pearson’s correlation (r). Data are presented as mean ±1 SD. In this study, the activity of the northern bat is shown as recorded by the three techniques, both grouped and for individual roosts.

## Data Availability

The datasets used and analyzed during the current study are available from the corresponding author on reasonable request.
